# Upper extremity disability and quality of life after breast cancer treatment in the Greater Plains Collaborative clinical research network

**DOI:** 10.1007/s10549-019-05184-1

**Published:** 2019-03-09

**Authors:** Elizabeth A. Chrischilles, Danielle Riley, Elena Letuchy, Linda Koehler, Joan Neuner, Cheryl Jernigan, Brian Gryzlak, Neil Segal, Bradley McDowell, Brian Smith, Sonia L. Sugg, Jane M. Armer, Ingrid M. Lizarraga

**Affiliations:** 1 University of Iowa College of Public Health, Iowa City, IA USA; 2 University of Iowa Holden Comprehensive Cancer Center, Iowa City, IA USA; 3 University of Minnesota, Minneapolis, MN USA; 4 Medical College of Wisconsin, Milwaukee, WI USA; 5 University of Kansas Medical Center, Kansas City, KS USA; 6 University of Iowa Carver College of Medicine, Iowa City, IA USA; 7 University of Missouri Sinclair School of Nursing, Columbia, MO USA; 8 College of Public Health, University of Iowa, S424 CPHB, 145 N. Riverside Dr., Iowa City, IA 52242-2007 USA

**Keywords:** Breast neoplasms, Shoulder pathology, Arm pathology, Quality of life, Rehabilitation

## Abstract

**Purpose:**

Chronic upper extremity disability (UED) is common after breast cancer treatment but under-identified and under-treated. Although UED has been linked to quality of life (QoL), the role of UED as mediator between contemporary treatment practices and QoL has not been quantified. This investigation describes UED in a contemporary sample of breast cancer patients and examines its relationship with personal and treatment factors and QoL.

**Methods:**

Eight hundred and thirty-three women diagnosed at eight medical institutions during 2013–2014 with microscopically confirmed ductal carcinoma in situ or invasive stage I–III breast cancer were surveyed an average of 22 months after diagnosis. UED was measured with a modified QuickDASH and QoL with the FACT-B. The questionnaire also collected treatments, sociodemographic information, comorbidity, body mass index, and a 3-item health literacy screener.

**Results:**

Women who received post-mastectomy radiation and chemotherapy experienced significantly worse UED and QoL. Women who had lower income, lower health literacy and prior diabetes, arthritis or shoulder diagnoses had worse UED. Patients with worse UED reported significantly worse QoL. Income and health literacy were independently associated with QoL after adjustment for UED but treatment and prior conditions were not, indicating mediation by UED. UED mediated 52–79% of the effect of mastectomy-based treatments on QoL as compared with unilateral mastectomy without radiation. UED and QoL did not differ by type of axillary surgery or post-mastectomy reconstruction.

**Conclusions:**

A large portion of treatment effect on QoL is mediated by UED. Rehabilitation practices that prevent and alleviate UED are likely to improve QoL for breast cancer survivors.

## Introduction

Chronic upper extremity disability (UED) is one of the most troublesome long-term complications of breast cancer treatment [1–3]. Persistent arm and shoulder impairments, defined as restricted shoulder mobility, lymphedema, and arm/shoulder pain, occur in 30–50% of breast cancer survivors [4]. It is now well established that breast cancer survivors have a high prevalence of arm/shoulder impairments that may persist for many years, and are associated with long-term activity limitations, participation restriction, and general quality of life (QoL) impact [5]. Most studies have been limited to lymphedema, even though long-lasting post-operative pain and problems with shoulder joint mobility may be as frequent or disabling [6–8].

While the prevalence of long-term UED has been well documented, screening for these problems has not become a routine part of survivorship care and physical impairments and activity limitations are under-identified and -treated [9]. Recent survivorship care guidelines have begun to recommend referral for these problems once they develop [10] and prospective surveillance is being advocated to identify and treat problems early rather than waiting for them to become more pronounced [11].

Upper extremity morbidity is modifiable, but not without cost and effort; thus, data quantifying its role in explaining effects of contemporary treatment practices on QoL are needed. Although QoL and upper extremity morbidity are increasingly examined as outcomes in breast cancer studies [8, 12–15], the potential mediating effect of UED on the QoL effects of contemporary treatment practice has not been quantified.

Studies of treatment factors related to upper extremity morbidity have largely focused on comparing different types of axillary or breast surgery. However, the profile of the breast cancer patient has changed over the last 10 years. Axillary dissection is less common and the rate of mastectomy, in particular bilateral mastectomy, is rising [16, 17]. Post-mastectomy reconstruction is also increasing at variable rates across the country, as is the use of post-mastectomy radiation [18]. The impact of these modern treatment trends on the incidence and severity of upper extremity morbidity is as yet poorly studied.

The objective of this paper is to describe the relationship of modern treatment characteristics with QoL in a contemporary sample of breast cancer patients and quantify the potential mediating effect of UED on this relationship. Because a variety of patient factors were expected to directly affect both upper extremity morbidity and QoL and must be accounted for, a secondary objective was to describe these relationships. For this study, we analyzed questionnaire and linked cancer registry data from the Share Thoughts on Breast Cancer Study, a project conducted within the Greater Plains Collaborative (GPC) Clinical Research Network (CRN) [19]. The GPC is one of 13 CRNs in PCORNet, the National Patient-Centered Clinical Research Network sponsored by the Patient-Centered Outcomes Research Institute.

## Methods

The study protocol was approved by the University of Iowa Institutional Review Board (IRB). The IRBs for the following collaborating medical centers ceded IRB review to the University of Iowa IRB pursuant to the GPC reliance agreement: University of Texas Southwestern Medical Center; University of Kansas Medical Center; University of Wisconsin Carbone Cancer Center; University of Nebraska Medical Center; University of Minnesota; Medical College of Wisconsin; and Marshfield Clinic Research Foundation. Informed consent was obtained from all individual participants included in the study. The datasets generated and analyzed during the current study are not publicly available because patients were explicitly asked whether they consented to re-use of their de-identified data by other unaffiliated investigators and this dataset includes patients who did not consent to re-use. A subset including only those with re-use consent can be provided by the corresponding author on reasonable request.

### Study population

Each participating medical center extracted, transformed, and loaded North American Association of Central Cancer Registries (NAACCR)-formatted data from their institution’s cancer registry into its i2b2 (Informatics for Integrating Biology and the Bedside) research warehouse. The GPC i2b2 research warehouse is fully de-identified with re-identification possible when accompanied by an approved IRB protocol [19].

From these data, each medical center ascertained all patients aged 18 or older diagnosed with breast cancer between January 2013 and May 2014. De-identified data files were submitted to the GPC Honest Broker who applied eligibility criteria and selected a random sample of 250 eligible patients from each center’s file. Eligible patients were women with microscopically confirmed ductal carcinoma in situ or invasive stage I-III breast cancer diagnosed during the study period. Women who were known to have been previously diagnosed with cancer per cancer registry records were excluded, as were women known to be deceased at the time the sample was selected. The sample of patients, plus a list of up to ten replacement patients, was provided to each center for re-identification and mailing. The replacement list was used in case a mailing was returned unopened or a patient was deceased. Two centers had fewer than 250 patients diagnosed during the study period.

### Data collection and management

All study materials were mailed in a single packet containing a cover letter from the participating medical center, a 21-page questionnaire, medical record consent, and $10 incentive. Questionnaires were mailed over a six-week period beginning June 19, 2015 and one re-mailing to non-respondents was conducted four weeks after the initial mailing. A total of 1,986 patients were invited and 1235 (62.2%) responded to a mailed questionnaire. Signed consent to obtain information from medical records was obtained for 852 (69%).

Study data were collected and managed using TeleForm and REDCap electronic data capture tools. TeleForm is a paper-based data capture software that uses recognition technology and REDCap (Research Electronic Data Capture) is a secure, web-based application [20] that was used for participation monitoring.

### Measures

Inclusion and exclusion criteria, tumor stage, and axillary surgery were NAACCR variables from the linked cancer registries. From the study questionnaire, we measured two of three World Health Organization (WHO) [21] International Classification of Functioning, Disability, and Health (ICF) components, impairment in body function and activity and participation, with nine items from the 11-item QuickDASH [22, 23] (two items were not included in the study questionnaire due to overlap with QoL measures). The test–retest reliability and validity of the QuickDASH has been demonstrated among breast cancer patients, including discriminating breast cancer survivors with frozen shoulder pain or upper extremity arthralgias [23]. QuickDASH is a short-form of the Disabilities of Arm, Shoulder, and Hand Questionnaire (DASH) [24, 25] and both QuickDASH and DASH have been mapped successfully to the ICF [26–28]. Questions include perceived difficulty with activities and severity of symptoms. Responses to each item were based on a 5-point Likert scale ranging from ‘No difficulty’ to ‘Unable (to do)’ or ‘No symptoms’ to ‘Extreme symptoms’. We created an overall score between 0 and 100 points by implementing the QuickDASH scoring method. As required by the scoring rule, no more than one missing item was allowed. Higher QuickDASH scores indicate more UED. We examined the performance of the 9-item measure against an 11-item measure obtained by incorporating the two overlapping items from the QoL measure (Appendix). Construct validity of the 9-item scale was also examined through factor analysis (Appendix) and comparison of scores for women with and without a baseline history of other arm or shoulder conditions. The Functional Assessment of Cancer Therapy for breast cancer (FACT-B) [29] was used to measure QOL. Higher FACT-B scores reflect better QoL.

The self-administered questionnaire was used to collect information on treatment characteristics, including primary surgery (unilateral mastectomy, bilateral mastectomy, lumpectomy), radiation (yes/no), chemotherapy (yes/no), endocrine therapy (yes/no), and reconstruction (implant, flap, none). To create treatment variables that reflected common treatment combinations, a factor analysis indicated that primary surgery and radiation could be combined (factor loadings > 0.8) and thus 5 categories were created (unilateral mastectomy without radiation, unilateral mastectomy with radiation, bilateral mastectomy without radiation, bilateral mastectomy with radiation, and lumpectomy). No lumpectomy without radiation category was created because 92% received radiation. In addition, self-reported sociodemographic data included age at diagnosis, race/ethnicity, highest level of completed education, insurance status at the time of diagnosis, marital status at the time of diagnosis, and annual household income. Body mass index (BMI) at time of diagnosis was calculated based on self-reported height and weight using the formula BMI = weight (kg)/height (m)^2^ and categorized as underweight (< 18.5 kg/m^2^), normal weight (18.5–24.9 kg/m^2^), overweight (25–29.9 kg/m^2^), and obese (30 + kg/m^2^). Health literacy was assessed using three self-reported items measuring how often: patients had someone help them read hospital materials, they were able to fill out medical forms alone (reverse-coded), and they had problems learning about their medical condition because of difficulty understanding written information [30]. Responses to each item were based on a 5-point Likert scale ranging from ‘Always’ to ‘Never’. This measure has previously been related to perceived care coordination among breast cancer patients [30] and validated against existing, longer health literacy assessments [31]. In keeping with prior use [30], a composite score was created by summing the scores of the three health literacy items. Categories for health literacy were assigned according to the following quartiles: low (first quartile, 0–12), medium (second and third quartile, 13–14), and high health literacy (fourth quartile, 15). Finally, self-reported information on co-morbidities diagnosed prior to breast cancer, included diabetes, arthritis, and rotator cuff, frozen shoulder, or other shoulder diagnoses.

### Statistical analysis

At the first stage of analysis, the distributions of continuous outcomes of interest (QuickDASH UED score and FACT-B QoL scale and sub-scales) were investigated. The distribution of QuickDASH scores was zero-inflated (19% of respondents reported a perfect 0 score indicating no difficulties) and left-skewed, while the FACT-B score distribution was somewhat right-skewed. We modeled the QuickDASH non-zero patients with a generalized linear model using a gamma distribution and a logit link function (SAS GENMOD procedure). Variable selection occurred in steps. The first step was the best sociodemographic model, step two added co-morbidity and retained the sociodemographic variables from the first step, and the third step added treatments. At this step, chemotherapy was so strongly associated with post-mastectomy radiation therapy that it could not be included as an independent variable and, for transparency, chemotherapy prevalence is henceforth reported wherever results for the combined surgery/radiation variable are discussed. These steps were repeated using logistic regression with a binary response variable indicating some vs. no difficulties. The corrected Akaike information criterion (AIC) was used to compare model fit. Least squares (LS) means on the original QuickDASH scale were calculated for each level of categorical independent variables, then corrected LS means were estimated, multiplying these values by the predicted probability of having a score above zero for each category based on the logistic regression model. Bootstrapping was used to find 95% confidence intervals for LS mean differences, comparing with a reference level.

A multiple regression model for FACT-B QoL with and without QuickDASH score as an independent variable and including other variables of interest was built the same way as the QuickDASH model. Residual diagnostics showed that it satisfied model assumptions using the original scale for both dependent variable (FACT-B QoL) and independent variable (QuickDASH score), so multiple linear regression was used for modeling (SAS GLM procedure). Candidate models were compared using coefficient of determination R-square.

We hypothesized that there was a causal relationship between treatment and FACT-B QoL and that QuickDASH score mediated this relationship. This was supported by the discovery that, after adjusting for covariates, there was a statistically significant association between the combined surgery/radiation treatment variable and FACT-B QoL and between this variable and QuickDASH score, and a strong association between QuickDASH score and FACT-B QoL. We thus proceeded with formal mediation analysis with multicategorical independent variables [32] to estimate the relative direct and indirect treatment effect on QoL and proportion of the total effect that was mediated.

## Results

Among the 852 questionnaire respondents who consented to medical record review, 835 had sufficiently complete responses to calculate valid FACT-B QoL and QuickDASH scores and two others were excluded from analysis due to missing age. These patients completed their questionnaires a mean (standard deviation, SD) of 22.1 (5.4) months after diagnosis. A description of the study population is in Table 1. Lumpectomy was performed for 56% of women and nearly 27% received bilateral mastectomy procedures. Almost all (92%) lumpectomy patients also reported receiving radiation therapy. Chemotherapy was administered to more than 90% of patients who received post-mastectomy radiation, and administered to 33% (unilateral mastectomy) to 48% (bilateral mastectomy) of patients who did not receive radiation. The mean (SD) QuickDASH score was 15.2 (16.11) and the Cronbach’s alpha coefficient was 0.89 (Appendix).

**Table 1:** Demographics and treatment characteristics of participants (*n* = 833)

Variable	*n* (%)
Age, in years	
<50	232 (27.9)
50–59	256 (30.7)
60–69	239 (28.7)
70+	106 (12.7)
Race/ethnicity^a^	
White	770 (92.4)
Black	27 (3.2)
Hispanic	18 (2.2)
Other	15 (1.8)
Annual household income, mean (SD)	$66,579 (28,371)
Highest level of education^a^	
Less than high school	17 (2.0)
High school graduate or G.E.D	137 (16.4)
Some college or 2-year degree	247 (29.7)
College graduate	230 (27.6)
More than a college degree	197 (23.6)
Insurance status^a^	
Insured (private +Medicare)	772 (92.7)
Any Medicaid	42 (5.0)
Uninsured	14 (1.7)
Marital status at diagnosis^a^	
Married/living with partner	618 (74.2)
Not married	214 (25.7)
BMI at diagnosis	
Underweight (< 18.5 kg/m^2^)	6 (0.7)
Normal weight (18.5–24.9 kg/m^2^)	319 (38.3)
Overweight (25.0-29.9 kg/m^2^)	229 (27.5)
Obese (30+ kg/m^2^)	279 (33.5)
Health literacy	
Low health literacy	198 (23.8)
Medium health literacy	271 (32.5)
High health literacy	364 (43.7)
AJCC stage at diagnosis	
0	134 (16.1)
I	377 (45.3)
II	229 (27.5)
III	75 (9.0)
Surgery and radiation treatment	
Unilateral mastectomy, no radiation (33% with chemotherapy)	99 (11.9)
Lumpectomy, 92% with radiation (36% with chemotherapy)	466 (55.9)
Bilateral mastectomy, no radiation (48% with chemotherapy)	156 (18.7)
Unilateral mastectomy, radiation (91% with chemotherapy)	46 (5.5)
Bilateral mastectomy, radiation (94% with chemotherapy)	66 (7.9)
Axillary surgery	
SLNB	505 (60.6)
ALND	254 (30.5)
None	74 (8.9)
Reconstruction	
Implant	180 (21.6)
Flap	69 (8.3)
None	584 (70.1)
Endocrine therapy	541 (64.9)
Prior rotator cuff/frozen shoulder	116 (13.9)
Prior arthritis	226 (27.1)
Prior diabetes	68 (8.2)
QuickDASH, mean (SD)	15.2 (16.1)
Fact-B, mean (SD)	115.0 (19.0)

*BMI* body mass index, *SLNB* sentinel lymph node biopsy, *ALND* axillary lymph node dissection

^a^Does not total 833 due to missing values

Table 2 displays associations of self-reported sociodemographic and treatment characteristics with UED. Women with less income, who had lower health literacy, or who had a history of diabetes, arthritis, or shoulder diagnoses, reported significantly more disability. After multivariable adjustment, the combined surgery/radiation treatment variable remained significantly associated with QuickDASH score. In particular, patients treated with post-mastectomy radiation (accompanied by chemotherapy in over 90% of cases) experienced the greatest (9 points) disability compared with the reference category, unilateral mastectomy without radiation (accompanied by chemotherapy in 33%). Age, body mass index, endocrine therapy, type of axillary surgery and reconstruction were not associated with QuickDASH score.

**Table 2:** Relationship of personal and treatment-related characteristics with upper extremity disability (QuickDASH total score) (*n* = 833)

Characteristics	*n*	Bivariable analysis		Multivariable analysis^a^	
		Mean (SD)	*p* value^c^	LS means (95% CI)	Difference in LS means (95% CI)
Age, in years			0.3170		
<50	232	13.6 (15.8)		24.14 (18.10, 30.77)	(Ref)
50–59	256	14.5 (15.1)		21.77 (16.38, 27.09)	− 2.37 (− 6.65, 1.59)
60–69	239	16.3 (17.4)		20.94 (15.54, 26.43)	− 3.20 (− 7.86, 1.22)
70+	106	17.9 (16.1)		20.44 (14.80, 27.01)	− 3.70 (− 8.92, 1.33)
Annual household income (continuous)^b^			< 0.0001		
At mean income				21.83 (16.70, 27.21)	
At mean income + $5000				21.13 (16.17, 26.32)	
BMI at diagnosis			0.7829		
Underweight	6	11.1 (8.4)		19.30 (8.94, 33.43)	− 2.27 (− 12.57, 11.35)
Normal weight	321	12.4 (14.5)		21.57 (17.63, 26.11)	(Ref)
Overweight	229	15.2 (15.8)		22.06 (18.13, 26.37)	0.49 (− 3.03, 4.14)
Obese	279	18.4 (17.7)		23.61 (19.61, 28.06)	2.04 (− 1.62, 5.62)
Health literacy			0.0062		
Low	199	18.4 (17.5)		25.18 (18.69, 32.30)	5.57 (1.67, 10.00)
Medium	271	15.2 (15.5)		20.89 (15.91, 26.25)	1.28 (− 1.59, 4.32)
High	365	13.4 (15.5)		19.61 (14.76, 24.47)	(Ref)
Surgery and radiation treatment			0.0093		
Unilateral mastectomy, no radiation (33% with chemotherapy)	99	14.0 (15.5)		17.00 (12.37, 22.34)	(Ref)
Lumpectomy, 92% with radiation (36% with chemotherapy)	467	14.3 (15.6)		18.71 (13.59, 23.75)	1.71 (− 2.16, 5.69)
Bilateral mastectomy, no radiation (48% with chemotherapy)	157	14.8 (16.1)		22.41 (16.63, 28.53)	5.41 (0.73, 10.56)
Unilateral mastectomy, radiation (91% with chemotherapy)	46	22.4 (20.7)		25.81 (16.42, 36.29)	8.82 (0.84, 17.59)
Bilateral mastectomy, radiation (94% with Chemotherap)y	66	18.8 (15.2)		25.89 (18.74, 33.91)	8.90 (2.80, 15.80)
Axillary surgery			0.5150		
SLNB	506	14.0 (16.2)		21.99 (16.76, 27.89)	2.28 (− 2.62, 7.01)
ALND	255	18.1 (15.9)		23.65 (18.36, 29.10)	3.94 (− 1.48, 9.01)
None	74	13.1 (15.0)		19.71 (13.40, 26.48)	(Ref)
Reconstruction			0.5069		
Implant	181	12.6 (13.9)		19.95 (14.35, 25.69)	− 2.12 (− 6.72, 2.45)
Flap	70	17.7 (17.6)		23.39 (16.54, 31.22)	1.32 (− 4.56, 7.56)
None	584	15.7 (16.5)		22.07 (16.92, 27.59)	(Ref)
Endocrine therapy			0.3899		
Yes	543	15.1 (15.4)		21.34 (16.49, 26.76)	− 0.48 (− 4.02, 2.26)
No	292	15.2 (17.4)		22.18 (16.58, 28.04)	(Ref)
Prior rotator cuff/frozen shoulder			0.0020		
Yes	116	22.9 (19.0)		25.17 (18.77, 32.10)	6.57 (2.18, 11.24)
No	719	13.9 (15.2)		18.60 (13.78, 23.28)	(Ref)
Prior arthritis			< 0.0001		
Yes	227	22.2 (18.1)		25.78 (19.96, 32.06)	7.54 (3.96, 11.49)
No	608	12.6 (14.5)		18.24 (13.39, 23.33)	(Ref)
Prior diabetes			0.0249		
Yes	68	23.2 (19.3)		24.47 (17.74, 31.81)	5.11 (0.57, 10.31)
No	767	14.5 (15.6)		19.36 (14.99, 23.77)	(Ref)

*LS* least squares, *BMI* body mass index, *SLNB* sentinel lymph node biopsy, *ALND* axillary lymph node dissection

^a^Two-part model was used for QuickDASH score to account for relatively high percent of 0 scores and skewed distribution- logistic regression for QuickDASH score as dichotomous dependent variable (= 0 vs. >0) and Gamma regression for QuickDASH > 0 only with the same independent variables. Bootstrapping was performed to find least squares (LS) means and 95% CI: for each bootstrap sample Gamma regression LS Means are calculated and multiplied by predicted probability of QuickDASH > 0 from logistic regression model. Resampling with *N* = 1000/2000/3000 replications was tried out. Computational results stabilized after *N* > 2000, bootstrapping results with *N* = 3000 are reported

^b^For annual household Income (continuous), predicted probabilities and predicted QuickDASH score for mean value and mean value+$5000 (1 unit) of income were calculated from logistic regression and gamma regression. Adjusted predicted score was calculated by multiplying on predicted probability for QuickDASH > 0

^c^Tests for overall significance for dichotomous or multi-categorical independent variables (*χ*^2^ statistic *p* value)

The combined surgery/radiation treatment variable was also significantly associated with FACT-B QoL scores with post-mastectomy radiation treatment groups reporting substantially lower QoL (Table 3). Younger women, those with lower income, who had lower health literacy, or who had a history of arthritis, or shoulder diagnoses, also reported significantly lower QoL on the FACT-B measure, whereas body mass index, a history of diabetes, endocrine therapy, reconstruction, and type of axillary surgery were not associated with QoL. QuickDASH score was strongly associated with QoL. After the QuickDASH score was included in these models, age, income, and health literacy were still significantly associated with QoL, however, there remained no significant relationship between treatment or history of arthritis or shoulder diagnoses with QoL, indicating a mediating role of the QuickDASH score on their relationships with QoL.

**Table 3:** Relationship of personal and treatment-related characteristics with quality of life (FACT-B total score), with and without including upper extremity disability (QuickDASH total score) (*n* = 833)

Characteristics	Total fact-B multiple regression model without QuickDASH score (*R*^2^ = 0.17)			Total fact-B multiple regression model with QuickDASH score (*R*^2^ = 0.38)		
	Estimate (SE)	*p* value^a^	LS means (95% CI)^b^	Estimate (SE)	*p* value^a^	LS means (95% CI)^b^
Intercept	109.11 (3.95)	< 0.0001		117.65 (3.44)	< 0.0001	
QuickDASH score, continuous				− 0.60 (0.04)	< 0.0001	
Age, in years		< 0.0001			< 0.0001	
<50 (reference)			103.22 (98.04,108.40)			108.62 (104.11,113.13)
50–59	5.22 (1.69)	0.0021	108.44 (103.25,113.64)	4.43 (1.46)	0.0025	113.05 (108.54,117.56)
60–69	11.92 (1.86)	< 0.0001	115.14 (109.84,120.45)	10.63 (1.61)	< 0.0001	119.25 (114.65,123.85)
70+	15.16 (2.42)	< 0.0001	118.38 (112.41,124.36)	13.22 (2.09)	< 0.0001	121.84 (116.67,127.01)
Annual household income, continuous (unit value=$5000)	0.80 (0.12)	< 0.0001		0.49 (0.10)	< 0.0001	
BMI at diagnosis		0.5534			0.7531	
Underweight	− 1.16 (7.31)	0.8736	111.38 (96.66,126.10)	− 1.20 (6.31)	0.8487	115.34 (102.64,128.04)
Normal weight (reference)			112.54 (108.79,116.30)			116.55 (113.28,119.82)
Overweight	− 1.78 (1.56)	0.2555	110.77 (106.99,114.54)	− 1.46 (1.35)	0.2770	115.08 (111.79,118.38)
Obese	− 2.04 (1.55)	0.1881	110.50 (106.94,114.06)	− 0.76 (1.34)	0.5727	115.79 (112.66,118.92)
Health literacy		0.0003			0.0063	
Low	− 6.56 (1.61)	< 0.0001	107.71 (102.32,113.09)	− 4.44 (1.39)	0.0015	113.30 (108.61,117.99)
Medium	− 2.34 (1.42)	0.1003	111.92 (106.87,116.98)	− 1.70 (1.23)	0.1679	116.04 (111.65,120.42)
High (reference)			114.27 (109.19,119.34)			117.74 (113.34,122.13)
Surgery and radiation treatment		0.0233			0.5498	
Unilateral mastectomy, no radiation (33% with chemotherapy) (reference group)			114.80 (109.25,120.36)			117.12 (112.32,121.92)
Lumpectomy, 92% with radiation (36% with chemotherapy)	− 0.54 (2.26)	0.8115	114.26 (108.88,119.65)	− 0.52 (1.95)	0.7896	116.60 (111.95,121.25)
Bilateral mastectomy, no radiation (48% with chemotherapy)	− 2.61 (2.35)	0.2660	112.19 (106.82,117.55)	− 0.53 (2.03)	0.7952	116.59 (111.94,121.25)
Unilateral mastectomy, radiation (91% with chemotherapy)	− 6.13 (3.25)	0.0598	108.67 (101.59,115.75)	− 2.20 (2.81)	0.4351	114.92 (108.77,121.08)
Bilateral mastectomy, radiation (94% with chemotherapy)	− 8.23 (2.92)	0.0049	106.57 (100.14,113.00)	− 3.91 (2.53)	0.1228	113.21 (107.62,118.81)
Axillary surgery		0.0614			0.2294	
SLNB	− 4.78 (2.22)	0.0318	109.98 (105.01,114.96)	− 3.30 (1.92)	0.0863	114.47 (110.15,118.79)
ALND	− 5.62 (2.42)	0.0206	109.14 (104.12,114.17)	− 2.94 (2.10)	0.1614	114.83 (110.45,119.21)
None (reference)			114.77 (108.65,120.88)			117.77 (112.48,123.06)
Reconstruction		0.7332			0.9383	
Implant	1.29 (2.19)	0.5563	112.32 (106.90,117.74)	− 0.58 (1.90)	0.7617	115.29 (110.60,119.98)
Flap	− 0.49 (2.67)	0.8535	110.54 (104.21,116.86)	0.05 (2.30)	0.9810	115.92 (110.43,121.41)
None (reference)			111.03 (106.04,116.03)			115.86 (111.52,120.21)
Endocrine therapy						
Yes	0.12 (1.31)	0.9266	111.36 (106.42,116.30)	0.07 (1.13)	0.9502	115.73 (111.43,120.02)
No (reference)			111.24 (106.13,116.35)			115.66 (111.22,120.09)
Prior rotator cuff/frozen shoulder						
Yes	− 6.25 (1.84)	0.0007	108.17 (102.60,113.74)	− 1.98 (1.61)	0.2199	114.70 (109.84,119.56)
No (reference)			114.42 (109.65,119.19)			116.68 (112.56,120.80)
Prior arthritis						
Yes	− 4.32 (1.56)	0.0057	109.14 (103.96,114.31)	− 0.07 (1.37)	0.9607	115.66 (111.13,120.18)
No (reference)			113.46 (108.45,118.47)			115.72 (111.39,120.06)
Prior diabetes						
Yes	− 0.68 (2.32)	0.7702	110.96 (104.85,117.06)	2.27 (2.01)	0.2587	116.83 (111.52,122.14)
No (reference)			111.64 (107.13,116.14)			114.56 (110.66,118.45)

*BMI* body mass index, *SLNB* sentinel lymph node biopsy, *ALND* axillary lymph node dissection

^a^*p* values for tests for overall significance are reported for all independent variable, for multi-categorical independent variables *p* values for tests for comparison with reference level are also included (*p* values corresponding to each category in *p* value columns)

^b^LS means are least squares means (adjusted means) calculated from multiple linear regression model

The indirect effect of treatments and proportion of total effect of treatments mediated through QuickDASH is displayed in Table 4. We estimated the proportion mediated for individual treatment categories where QuickDASH score was found to mediate 52–79% of the total effect of mastectomy-based treatments as compared with unilateral mastectomy without radiation. The proportion mediated estimated for lumpectomy with radiation was small (4%), and should be interpreted with caution because there was only a very small total effect (− 0.54) compared with unilateral mastectomy without radiation.

**Table 4:** Mediation analysis results for FACT-B QoL versus treatment as causal variable and QuickDASH score as mediator

	Total effect (SE)^a^	Direct effect (SE)^b^	Relative indirect effect (95% CI)^c^	Proportion mediated^d^
Treatment (categorical)				
Unilateral mastectomy, no radiation (33% with chemotherapy)	(Ref)			
Lumpectomy, 92% with radiation (36% with chemotherapy)	− 0.54 (2.26)	− 0.52 (1.95)	− 0.02 (− 2.26, 2.29)	0.04
Bilateral mastectomy, no radiation (48% with chemotherapy)	− 2.61 (2.35)	− 0.53 (2.03)	− 2.08 (− 4.4, 0.14)	0.79
Unilateral mastectomy, radiation (91% with chemotherapy)	− 6.13 (3.25)	− 2.20 (2.81)	− 3.93 (− 8.21,− 0.18)	0.64
Bilateral mastectomy, radiation (94% with chemotherapy)	− 8.23 (2.92)	− 3.91 (2.53)	− 4.32 (− 7.44,− 1.57)	0.52

^a^Total effect parameter estimate is from FACT-B model without QuickDASH included

^b^Direct effect parameter estimate is from FACT-B model with QuickDASH included

^c^Calculated as total effect minus direct effect. Bootstrapping method with 5,000 replications is used to estimate 95% CI for relative indirect effect

^d^Calculated as indirect effect divided by total effect. On average the proportion mediated was < 0.8, so this is partial mediation

## Discussion

In this study, of 833 women with stage 0-III breast cancer diagnosed at eight large medical institutions in six states, the mean UED score was 50% higher than the mean (SD) of 10.1 (14.7) that was reported for a general population sample in one study [33]. Patients who received post-mastectomy radiation (13.4% of all patients) also received chemotherapy in over 90% of cases; these women experienced poorer QoL than other treatment groups, largely mediated through an adverse effect of treatment on UED. Women with low income and lower scores on a health literacy screening measure reported poorer outcomes, even after adjusting for other demographic and clinical characteristics.

The effect of type of breast surgery on upper arm morbidity has been studied extensively, with conflicting results [13, 15, 34–36]. We chose to examine combinations of surgery and radiation with respect to arm morbidity because the two modalities are closely linked, and also in light of the increasing use of post-mastectomy radiation treatment [37, 38]. We found that treatment including post-mastectomy radiation was strongly associated with UED as well as worse QoL, regardless of whether one or both breasts were removed, whereas treatment with lumpectomy and radiation was associated with comparable UED and QoL to that exhibited by patients who received unilateral mastectomy without radiation. The higher rates of chemotherapy in the post-mastectomy radiation treatment groups may be contributing to these differences [39], but also radiation after lumpectomy differs greatly from post-mastectomy radiation in its extent. Standard whole breast radiation involves two fields targeting the breast only, often including a boost to the tumor bed, whereas modern post-mastectomy radiation treatment includes treatment to the chest wall, infra- and supraclavicular area, posterior axilla and internal mammary nodes, with much greater potential for muscle and soft tissue fibrosis and loss of function [40–42].

Differences attributed to surgery and radiation treatment could occur through wound healing, surgical site infections, additional reconstruction and surgical procedures, and recovery time. Complications from additional treatments, and additive symptomatology including fibrosis, cording, neuropathy and lymphedema are all plausible explanations for the observed treatment effects on UED and QoL [43–46]. They may also have important psychologic or indirect effects. Since upper extremities are needed for so many daily activities, it would not be surprising that any symptoms are particularly intrusive [13], increasing their effect on QoL. The results of our mediation analysis showing that UED (QuickDASH) indeed explains a large portion of the effects of treatments on FACT-B QoL are consistent with this hypothesis.

Our study supports prior research [47] showing that cancer patients with low health literacy have lower QoL after adjusting for sociodemographic and clinical covariates. This suggests that health literacy may have a direct effect on UED and QoL outcomes among breast cancer survivors. Potential mechanisms may include effects on how patients access and use health care, patient-provider communication, and self-care knowledge and abilities [48]. Alternatively, our limited 3-item measure of health literacy is a screening tool for identifying patients with potentially inadequate health literacy and could be correlated with other unmeasured factors. A longer health literacy assessment instrument such as the Short Test of Functional Health Literacy in Adults [49] would provide more definitive assessment.

Neither axillary node dissection abstracted by tumor registries [50] nor self-reported post-mastectomy reconstruction were associated with UED or QoL. Axillary node dissection was performed in 30% of cases and our negative finding contrasts with two large randomized clinical trials and a number of other studies [12–14, 51, 52], but is consistent with two recent observational studies [34, 39]. Reconstruction was reported by 30% of our patients. Post-mastectomy reconstruction differs substantially from post-lumpectomy reconstruction; however, the null finding persisted when we excluded lumpectomy-treated patients and repeated the analyses (data not shown). The rate of post-mastectomy reconstruction has been rising [53] and there have been a few studies with mixed results [34, 54] of the effect on upper extremity morbidity. Future prospective research that collects more precise information on the type of reconstruction and subsequent outcomes appears warranted.

While the prevalence of UED has been reported previously in breast cancer survivors, this study quantified the large portion of treatment effect on QoL that is mediated by UED and lends support to calls for prospective surveillance and early detection of UED to address established [9] under-identification and under-treatment of these physical impairments and activity limitations. Previous research has demonstrated associations between upper-body morbidity and QoL in breast cancer patients [55]. Since upper body morbidity is modifiable, any intervention to improve it may positively impact QoL. Early physical therapy intervention, such as early mobility, range of motion exercises, manual therapy, lymphedema education, and/or scar management, have demonstrated a lower incidence in arm and shoulder morbidity and better QoL in patients following surgery for breast cancer [56–58]. Early diagnosis and treatment for lymphedema through a breast cancer rehabilitation surveillance program has been able to potentially reverse and reduce risk of chronic lymphedema onset [59] and analyses project the cost-effectiveness of such a model [60]. A supervised physical therapy program consisting of aerobic and resistance exercises improved cardiorespiratory fitness, strength, and QoL in women with early-stage breast cancer [61]. Since QoL can predict survival in women with breast cancer [62, 63], it is important to consider interventions, such as physical therapy, that address UED which can improve QoL and potentially survival. Other rehabilitation interventions may also include arm, shoulder, and neck range of motion and stretching, strengthening, postural education, counseling, and occupational therapy.

At this time, there is no standard model for rehabilitation follow-up of patients with early-stage breast cancer diagnoses. Guidelines have been created by the National Comprehensive Cancer Network and American Society of Clinical Oncology to address symptom-specific survivorship [64]. The results of our study support referring patients to a rehabilitation specialist. Findings also support assessment of patient health literacy during the initial clinical encounter to guide communications during diagnosis, treatment, and follow-up. These results highlight the importance of further patient-centered outcomes research to evaluate effectiveness of early identification and referral models [11].

Strengths of this study include high response rate, generalizability to breast cancer care in large medical centers in six different states, availability of linked cancer registry data, and use of validated measures of UED and cancer QoL. Limitations include general limitations of a cross-sectional design, use of self-reported data on treatments and reconstruction, and generalizability. With a cross-sectional design, it is possible that treatment groups varied in QoL and shoulder and arm function prior to their breast cancer. To minimize this concern, we were able to examine and control for a number of sociodemographic and clinical characteristics including history of arthritis, diabetes and shoulder diagnoses. In addition, the pattern of relationships are consistent with the hypothesized mechanism and with systematic reviews of longitudinal studies of range of motion and lymphedema [4, 5].

Because our treatment data were self-reported, we could not evaluate nuances of treatment, such as radiation dose or location or surgical techniques. We also did not investigate the mechanisms leading to UED, such as postoperative complications or specific shoulder and upper limb symptomatology. The dataset included 9 of the 11 original QuickDASH items. In analyses (Table 2) comparing scores for women with and without a baseline history of other arm or shoulder conditions, the validity of the shortened version was supported and the score distribution, Cronbach’s alpha reliability, and factor analysis results were comparable to an 11-item version derived by including comparable items from the study QoL questionnaire (Appendix). Participants were surveyed an average of 22 months following diagnosis of cancer, so our findings may not generalize to longer-term outcomes. In prior studies [12, 13, 52], chronic arm morbidity following breast cancer surgery was greatest 1 year following surgery and declined over the years following. Our study covered care delivered in eight academic and large community medical institutions in six states. However, this may not be representative of treatment in other care settings.

In summary, this study assessed the effects of contemporary treatment practices on UED and QoL. Negative effects of primary breast cancer treatment, including mastectomy, post-mastectomy radiation therapy, and chemotherapy on QoL were largely mediated through effects on UED. The magnitude of the effect, coupled with availability of effective rehabilitation practices, underscores the unmet need and the large opportunity to improve QoL for breast cancer survivors.

## Appendix

### Performance of the shortened (9-item) form of the QuickDash in the Share Thoughts on Breast Cancer Study

#### Overview

From the *Share Thoughts on Breast Cancer Study* questionnaire, upper extremity disability was measured with nine items from the 11-item QuickDASH [22, 23]. Two items, interference with social activities and limitation in regular daily activities, were not included in the study questionnaire due to overlap with items in the Functional Assessment of Cancer Therapy for breast cancer (FACT-B) Quality of Life (QoL) measure.

In this Appendix, we examine the performance of the 9-item measure against an 11-item measure obtained by incorporating the two overlapping items from the QoL measure. Construct validity of the 9-item scale was also examined through factor analysis and comparison of scores for women with and without a baseline history of other arm or shoulder conditions (Table 2 in manuscript). Overall, these analyses confirm that the distribution for the 9-item scale and 11-item scale are quite similar and examination of Cronbach alpha with deleted variables did not show significant increase or decrease in the standardized alpha coefficients. Further, we illustrate through factor analysis that a one-factor solution is appropriate for both the 9-item and 11-item scale with the one factor solution explaining 92% and 91% of the variance, respectively. Women who had a history of arthritis, or shoulder diagnoses reported significantly more disability, with adjusted mean scores 7.5 and 6.5 points higher than women without these conditions.

#### Scoring and Cronbach’s alpha reliability of the study 9-item QuickDASH measure

Study QuickDASH definition—uses 9 of 11 original items, calculated as follows:

QuickDASHScore = ((QuickDASHSum/QuickDashCount) − 1)*25,

where QuickDASHSum = QuickDASHSumNew = SUM(intOpenJar, intHeavyChores, intCarryBag, intWashBack,intCutFood, intRecreationalActivity, intSleepingPain, intArmPain, intArmTingling).

QuickDashCount-number of non-missing among 9 items, only 1 missing item allowed for scale to be defined.

Cronbach coefficient alpha:

**Table:**

Variables	Alpha
Raw	0.876
Standardized	0.880

Cronbach coefficient alpha with deleted variable:

**Table:**

Deleted variable	Raw correlation	Raw alpha	Standardized correlation	Standardized alpha
intOpenJar	0.678	0.857	0.669	0.863
intHeavyChores	0.677	0.858	0.661	0.863
intCarryBag	0.676	0.859	0.662	0.863
intWashBack	0.646	0.860	0.642	0.865
intCutFood	0.496	0.875	0.501	0.877
intRecreationalActivity	0.738	0.851	0.730	0.857
intSleepingPain	0.572	0.867	0.581	0.870
intArmPain	0.646	0.860	0.660	0.863
intArmTingling	0.494	0.873	0.511	0.876

#### Selected factor analysis results for the study 9-item QuickDASH measure

Note: only the first eigenvalue (below) is greater than 1 and it is much larger than the second in value, so a 1-factor solution is appropriate; total variance = 4.46,1 factor solution explains 92% of variance.

First two eigenvalues:

**Table:**

Number	Eigenvalue
1	4.137
2	0.757

Note: Factor loadings (below) are high (usually 0.35–0.40 as threshold to belong to factor)

Factor loadings:

**Table:**

Variable	Factor1
intOpenJar	0.713
intHeavyChores	0.736
intCarryBag	0.721
intWashBack	0.699
intCutFood	0.525
intRecreationalActivity	0.796
intSleepingPain	0.614
intArmPain	0.709
intArmTingling	0.536

#### Scoring and Cronbach’s alpha reliability of a proxy 11-item QuickDASH measure

Proxy 11-item QuickDASH Definition—uses 9 of 11 original items, plus 2 items similar to the excluded items calculated as follows:

QuickDASHScore11 = ((QuickDASHSum/QuickDashCount) − 1)*25,

where QuickDASHSum = QuickDASHSumNew = SUM(intOpenJar, intHeavyChores, intCarryBag, intWashBack,intCutFood, intRecreationalActivity, intSleepingPain, intArmPain, intArmTingling, *inv_intAbleToWork, intLeisure*,).

QuickDashCount-number of non-missing among 11 items, only 1 missing item allowed for scale to be defined.

**Table:**

Standard QuickDASH items that were excluded from questionnaire	Questionnaire items substituted from FACT-B
During the past week, to what extent has your arm, shoulder or hand problem interfered with your normal social activities with family, friends, neighbours or groups? (Not at all, slightly, moderately, quite a bit, extremely)	From FACT-B social wellbeing domain—in the past 7 days….. I had trouble doing all of my regular leisure activities with others (Not at all, a little bit, somewhat, quite a bit, very much)
During the past week, were you limited in your work or other regular daily activities as a result of your arm, shoulder or hand problem? (Not limited at all, slightly limited, moderately limited, very limited, unable)	From FACT-B functional wellbeing domain—in the past 7 days… I am able to work (include work at home) (not at all, a little bit, somewhat, quite a bit, very much) (reversed to have the same direction as QuickDASH questions)

Note: The Cronbach’s alpha for the 11-item measure (below) was similar to the 9-item measure (0.89 below vs. 0.88 above)

Cronbach coefficient alpha:

**Table:**

Variables	Alpha
Raw	0.887
Standardized	0.891

Note: Examination of Cronbach alpha with deleted variables (below) did not show significant increase or decrease in the standardized alpha coefficients when either of the 2 FACT-B QoL items were deleted

Cronbach coefficient alpha with deleted variable:

**Table:**

Deleted variable	Raw correlation	Raw alpha	Standardized correlation	Standardized alpha
intOpenJar	0.663	0.873	0.662	0.878
intHeavyChores	0.716	0.870	0.701	0.875
intCarryBag	0.696	0.873	0.686	0.876
intWashBack	0.649	0.874	0.649	0.879
intCutFood	0.503	0.885	0.510	0.887
intRecreationalActivity	0.752	0.867	0.748	0.872
intSleepingPain	0.545	0.881	0.556	0.884
intArmPain	0.612	0.877	0.628	0.880
intArmTingling	0.479	0.884	0.496	0.888
inv_intAbleToWork	0.583	0.878	0.572	0.883
intLeisure	0.540	0.883	0.535	0.886

#### Selected factor analysis results for the proxy 11-item QuickDASH measure

Note: only the first eigenvalue (below) is greater than 1 and it is much larger than the second in value, so a 1-factor solution is appropriate; total variance = 5.32, 1 factor solution explains 91% of variance.

First 2 eigenvalues:

**Table:**

Number	Eigenvalue
1	4.831
2	0.876

Note: Factor loadings (below) are high (usually 0.35–0.40 as threshold to belong to factor).

Factor loadings:

**Table:**

Variable	Factor1
intOpenJar	0.704
intHeavyChores	0.770
intCarryBag	0.742
intWashBack	0.706
intCutFood	0.530
intRecreationalActivity	0.805
intSleepingPain	0.583
intArmPain	0.672
intArmTingling	0.516
inv_intAbleToWork	0.625
intLeisure	0.562

#### Description of study 9-item QuickDASH and proxy 11-item QuickDASH score distributions

Note: Distributions for the two scales (below) are not very different. There is a somewhat higher % of 0 for the 9-item scale.

**Table:**

Varname	Mean (SD)	Median (range)	Interquartile range	% (= 0)
QuickDASHScore	15.18 (16.12)	11.11 (0.00,86.11)	(2.78,22.22)	19.8
QuickDASHScore11	15.60 (16.01)	11.36 (0.00,86.36)	(4.55,22.73)	15.3
